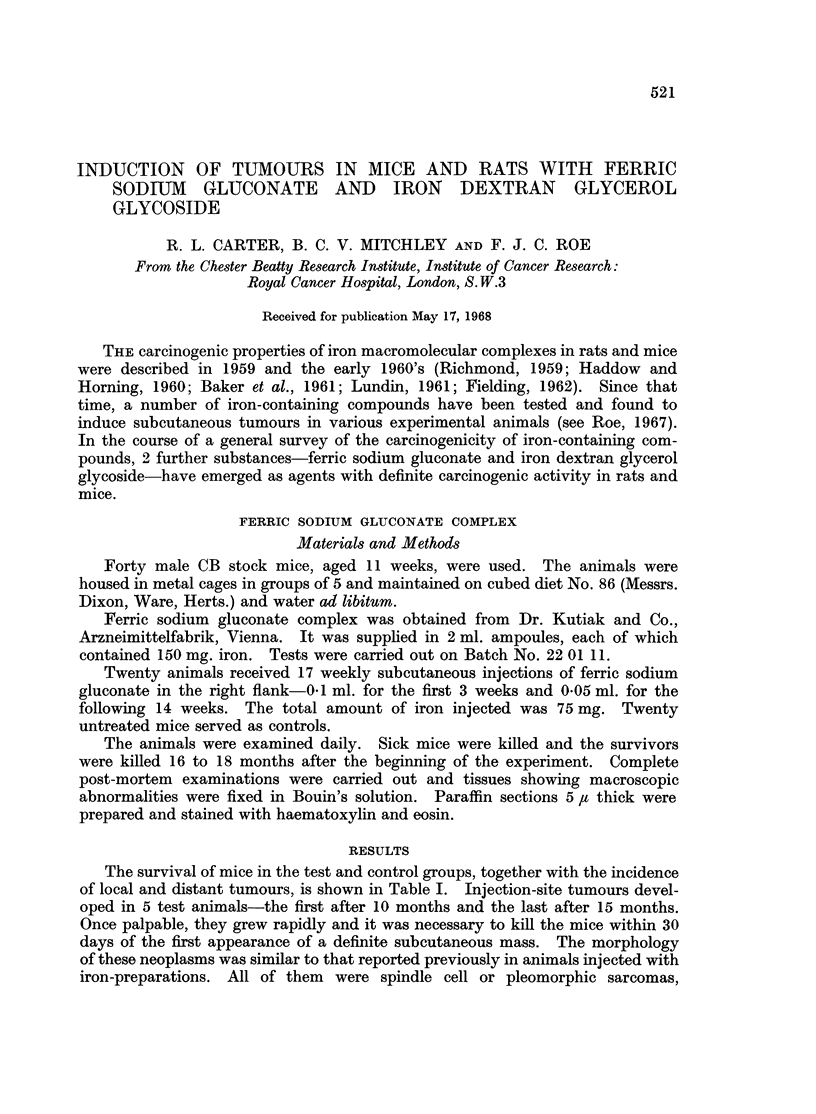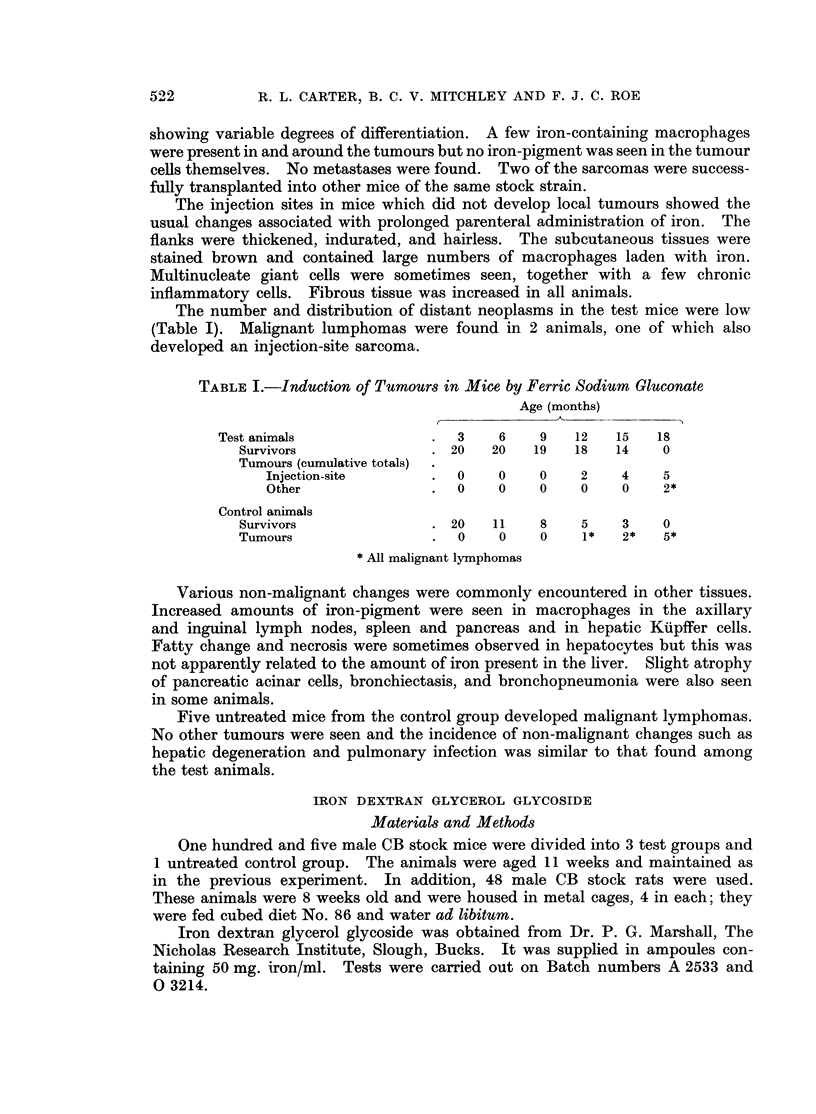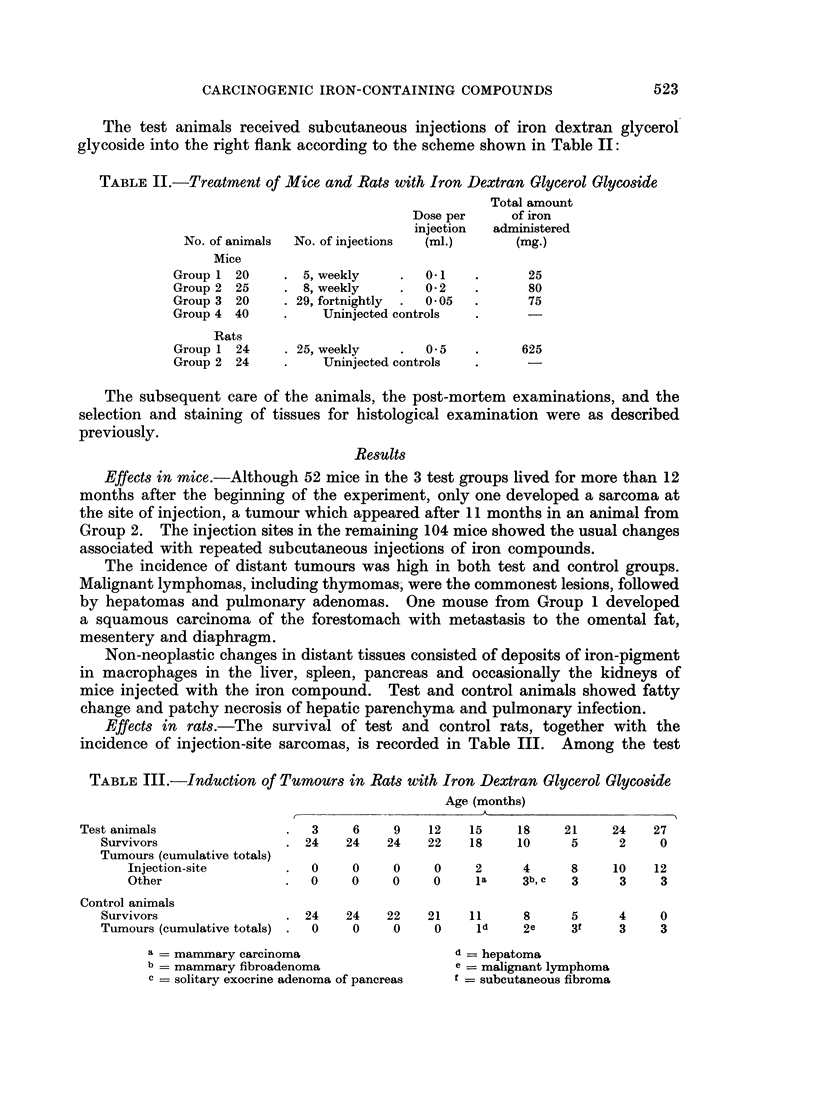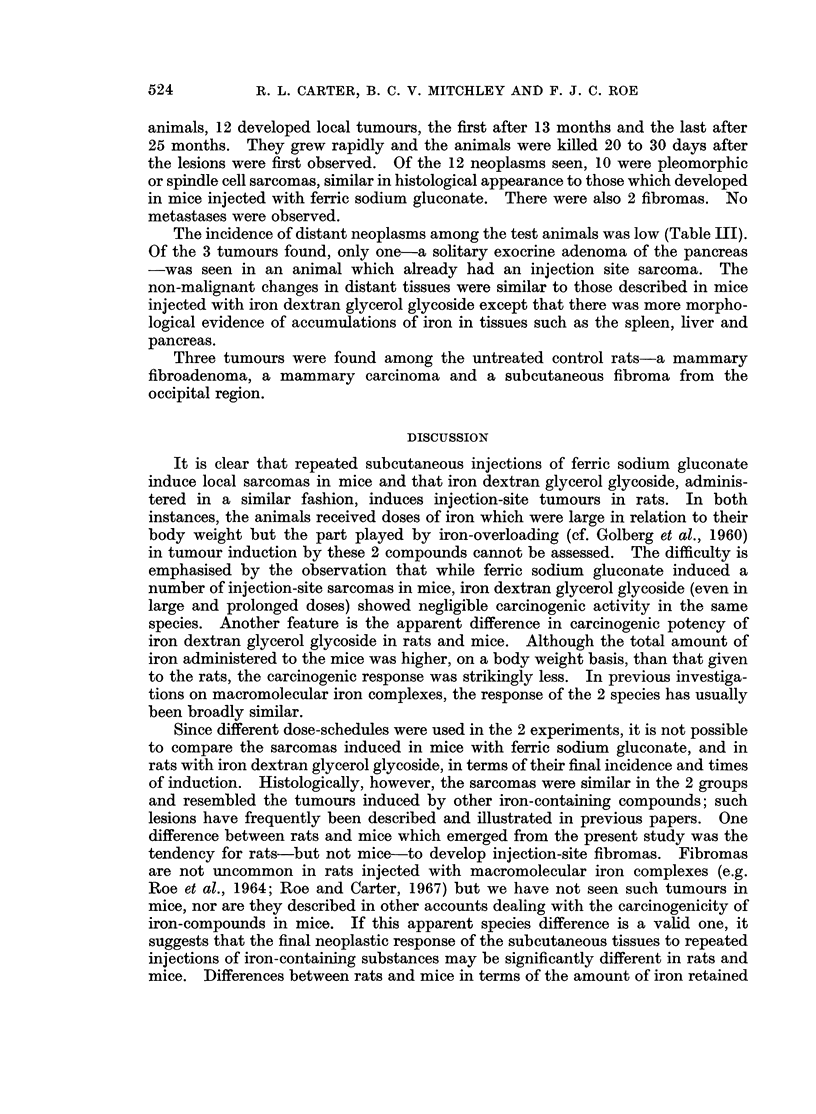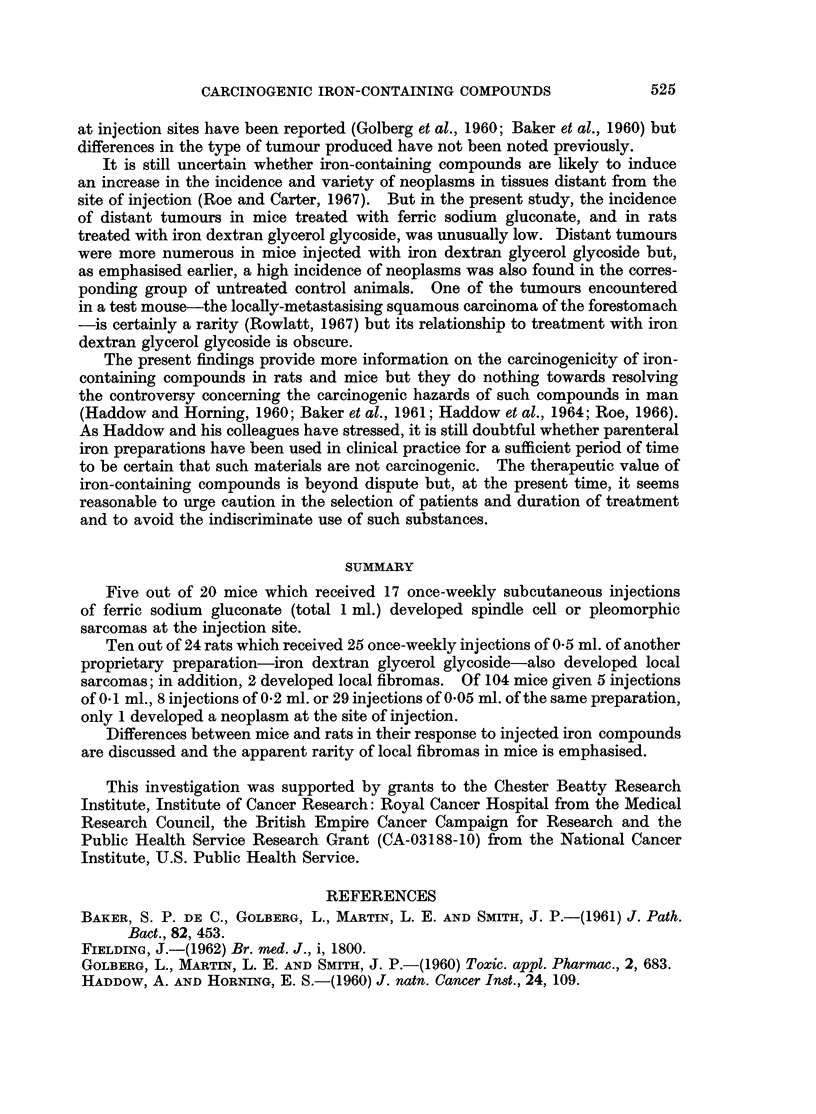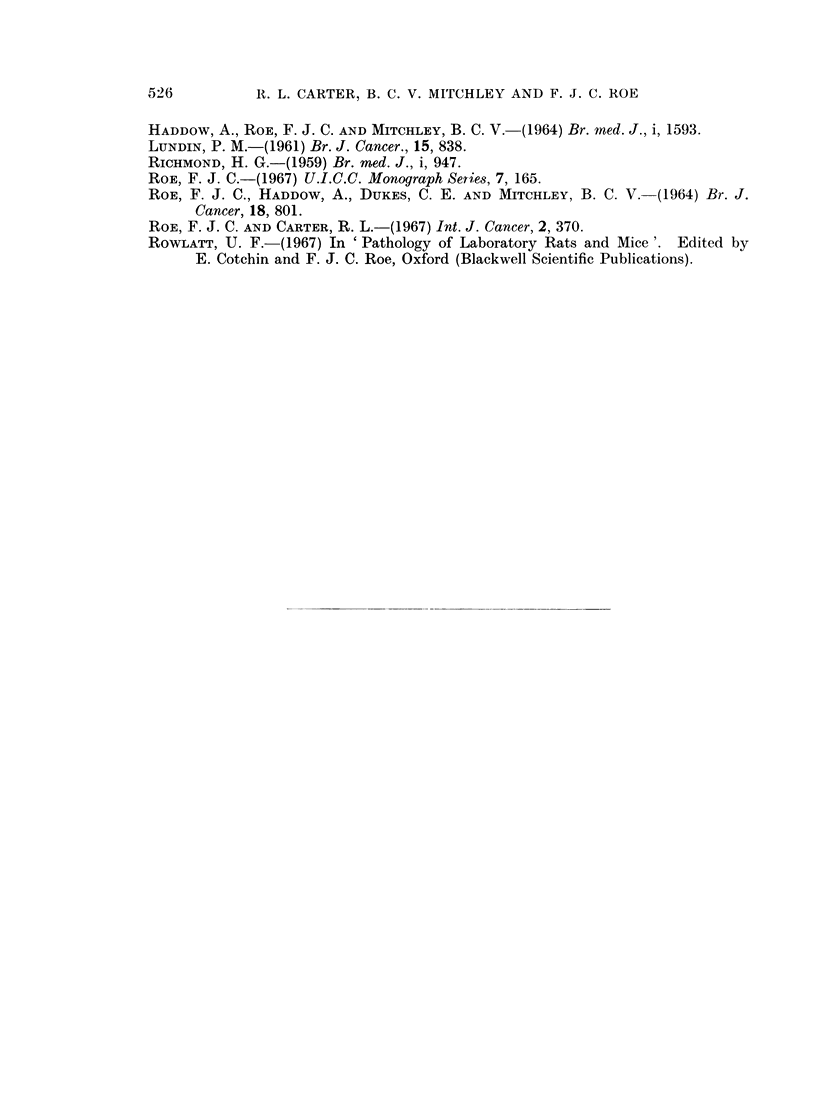# Induction of tumours in mice and rats with ferric sodium gluconate and iron dextran glycerol glycoside.

**DOI:** 10.1038/bjc.1968.62

**Published:** 1968-09

**Authors:** R. L. Carter, B. C. Mitchley, F. J. Roe


### INDUCTION OF TUMOURS IN MICE AND RATS WITH FERRIC SODIUM GLUCONATE AND IRON DEXTRAN GLYCEROL GLYCOSIDE

R. L. CARTER, B. C. V. MITCHLEY AND F. J. C. ROE

*From the Chester Beatty Research Institute, Institute of Cancer Research: Royal Cancer Hospital, London, S.W.3*

Received for publication May 17, 1968

THE carcinogenic properties of iron macromolecular complexes in rats and mice were described in 1959 and the early 1960's (Richmond, 1959; Haddow and Horning, 1960; Baker *et al.*, 1961; Lundin, 1961; Fielding, 1962). Since that time, a number of iron-containing compounds have been tested and found to induce subcutaneous tumours in various experimental animals (see Roe, 1967). In the course of a general survey of the carcinogenicity of iron-containing compounds, 2 further substances—ferric sodium gluconate and iron dextran glycerol glycoside—have emerged as agents with definite carcinogenic activity in rats and mice.

### FERRIC SODIUM GLUCONATE COMPLEX

### *Materials and Methods*

Forty male CB stock mice, aged 11 weeks, were used. The animals were housed in metal cages in groups of 5 and maintained on cubed diet No. 86 (Messrs. Dixon, Ware, Herts.) and water *ad libitum.*

Ferric sodium gluconate complex was obtained from Dr. Kutiak and Co., Arzneimittelfabrik, Vienna. It was supplied in 2 ml. ampoules, each of which contained 150 mg. iron. Tests were carried out on Batch No. 22 01 11.

Twenty animals received 17 weekly subcutaneous injections of ferric sodium gluconate in the right flank—0·1 ml. for the first 3 weeks and 0·05 ml. for the following 14 weeks. The total amount of iron injected was 75 mg. Twenty untreated mice served as controls.

The animals were examined daily. Sick mice were killed and the survivors were killed 16 to 18 months after the beginning of the experiment. Complete post-mortem examinations were carried out and tissues showing macroscopic abnormalities were fixed in Bouin's solution. Paraffin sections 5 $\mu$ thick were prepared and stained with haematoxylin and eosin.

### RESULTS

The survival of mice in the test and control groups, together with the incidence of local and distant tumours, is shown in Table I. Injection-site tumours developed in 5 test animals—the first after 10 months and the last after 15 months. Once palpable, they grew rapidly and it was necessary to kill the mice within 30 days of the first appearance of a definite subcutaneous mass. The morphology of these neoplasms was similar to that reported previously in animals injected with iron-preparations. All of them were spindle cell or pleomorphic sarcomas, showing variable degrees of differentiation. A few iron-containing macrophages were present in and around the tumours but no iron-pigment was seen in the tumour cells themselves. No metastases were found. Two of the sarcomas were successfully transplanted into other mice of the same stock strain.

The injection sites in mice which did not develop local tumours showed the usual changes associated with prolonged parenteral administration of iron. The flanks were thickened, indurated, and hairless. The subcutaneous tissues were stained brown and contained large numbers of macrophages laden with iron. Multinucleate giant cells were sometimes seen, together with a few chronic inflammatory cells. Fibrous tissue was increased in all animals.

The number and distribution of distant neoplasms in the test mice were low (Table I). Malignant lumphomas were found in 2 animals, one of which also developed an injection-site sarcoma.

TABLE I.—*Induction of Tumours in Mice by Ferric Sodium Gluconate*

| | Age (months) | | | | | |
|---|---|---|---|---|---|---|
| Test animals | 3 | 6 | 9 | 12 | 15 | 18 |
| Survivors | 20 | 20 | 19 | 18 | 14 | 0 |
| Tumours (cumulative totals) | | | | | | |
| Injection-site | 0 | 0 | 0 | 2 | 4 | 5 |
| Other | 0 | 0 | 0 | 0 | 0 | 2* |
| Control animals | | | | | | |
| Survivors | 20 | 11 | 8 | 5 | 3 | 0 |
| Tumours | 0 | 0 | 0 | 1* | 2* | 5* |

* All malignant lymphomas

Various non-malignant changes were commonly encountered in other tissues. Increased amounts of iron-pigment were seen in macrophages in the axillary and inguinal lymph nodes, spleen and pancreas and in hepatic Küpffer cells. Fatty change and necrosis were sometimes observed in hepatocytes but this was not apparently related to the amount of iron present in the liver. Slight atrophy of pancreatic acinar cells, bronchiectasis, and bronchopneumonia were also seen in some animals.

Five untreated mice from the control group developed malignant lymphomas. No other tumours were seen and the incidence of non-malignant changes such as hepatic degeneration and pulmonary infection was similar to that found among the test animals.

### IRON DEXTRAN GLYCEROL GLYCOSIDE

### *Materials and Methods*

One hundred and five male CB stock mice were divided into 3 test groups and 1 untreated control group. The animals were aged 11 weeks and maintained as in the previous experiment. In addition, 48 male CB stock rats were used. These animals were 8 weeks old and were housed in metal cages, 4 in each; they were fed cubed diet No. 86 and water *ad libitum*.

Iron dextran glycerol glycoside was obtained from Dr. P. G. Marshall, The Nicholas Research Institute, Slough, Bucks. It was supplied in ampoules containing 50 mg. iron/ml. Tests were carried out on Batch numbers A 2533 and O 3214.

The test animals received subcutaneous injections of iron dextran glycerol glycoside into the right flank according to the scheme shown in Table II:

TABLE II.—*Treatment of Mice and Rats with Iron Dextran Glycerol Glycoside*

| No. of animals | | No. of injections | Dose per injection (ml.) | Total amount of iron administered (mg.) |
|---|---|---|---|---|
| Mice | | | | |
| Group 1 | 20 | 5, weekly | 0·1 | 25 |
| Group 2 | 25 | 8, weekly | 0·2 | 80 |
| Group 3 | 20 | 29, fortnightly | 0·05 | 75 |
| Group 4 | 40 | Uninjected controls | | — |
| Rats | | | | |
| Group 1 | 24 | 25, weekly | 0·5 | 625 |
| Group 2 | 24 | Uninjected controls | | — |

The subsequent care of the animals, the post-mortem examinations, and the selection and staining of tissues for histological examination were as described previously.

### *Results*

*Effects in mice.*—Although 52 mice in the 3 test groups lived for more than 12 months after the beginning of the experiment, only one developed a sarcoma at the site of injection, a tumour which appeared after 11 months in an animal from Group 2. The injection sites in the remaining 104 mice showed the usual changes associated with repeated subcutaneous injections of iron compounds.

The incidence of distant tumours was high in both test and control groups. Malignant lymphomas, including thymomas, were the commonest lesions, followed by hepatomas and pulmonary adenomas. One mouse from Group 1 developed a squamous carcinoma of the forestomach with metastasis to the omental fat, mesentery and diaphragm.

Non-neoplastic changes in distant tissues consisted of deposits of iron-pigment in macrophages in the liver, spleen, pancreas and occasionally the kidneys of mice injected with the iron compound. Test and control animals showed fatty change and patchy necrosis of hepatic parenchyma and pulmonary infection.

*Effects in rats.*—The survival of test and control rats, together with the incidence of injection-site sarcomas, is recorded in Table III. Among the test

TABLE III.—*Induction of Tumours in Rats with Iron Dextran Glycerol Glycoside*

| | Age (months) | | | | | | | | |
|---|---|---|---|---|---|---|---|---|---|
| Test animals | 3 | 6 | 9 | 12 | 15 | 18 | 21 | 24 | 27 |
| Survivors | 24 | 24 | 24 | 22 | 18 | 10 | 5 | 2 | 0 |
| Tumours (cumulative totals) | | | | | | | | | |
| Injection-site | 0 | 0 | 0 | 0 | 2 | 4 | 8 | 10 | 12 |
| Other | 0 | 0 | 0 | 0 | 1[a] | 3[b, c] | 3 | 3 | 3 |
| Control animals | | | | | | | | | |
| Survivors | 24 | 24 | 22 | 21 | 11 | 8 | 5 | 4 | 0 |
| Tumours (cumulative totals) | 0 | 0 | 0 | 0 | 1[d] | 2[e] | 3[f] | 3 | 3 |

[a] = mammary carcinoma

[b] = mammary fibroadenoma

[c] = solitary exocrine adenoma of pancreas

[d] = hepatoma

[e] = malignant lymphoma

[f] = subcutaneous fibroma animals, 12 developed local tumours, the first after 13 months and the last after 25 months. They grew rapidly and the animals were killed 20 to 30 days after the lesions were first observed. Of the 12 neoplasms seen, 10 were pleomorphic or spindle cell sarcomas, similar in histological appearance to those which developed in mice injected with ferric sodium gluconate. There were also 2 fibromas. No metastases were observed.

The incidence of distant neoplasms among the test animals was low (Table III). Of the 3 tumours found, only one—a solitary exocrine adenoma of the pancreas —was seen in an animal which already had an injection site sarcoma. The non-malignant changes in distant tissues were similar to those described in mice injected with iron dextran glycerol glycoside except that there was more morphological evidence of accumulations of iron in tissues such as the spleen, liver and pancreas.

Three tumours were found among the untreated control rats—a mammary fibroadenoma, a mammary carcinoma and a subcutaneous fibroma from the occipital region.

### DISCUSSION

It is clear that repeated subcutaneous injections of ferric sodium gluconate induce local sarcomas in mice and that iron dextran glycerol glycoside, administered in a similar fashion, induces injection-site tumours in rats. In both instances, the animals received doses of iron which were large in relation to their body weight but the part played by iron-overloading (cf. Golberg *et al.*, 1960) in tumour induction by these 2 compounds cannot be assessed. The difficulty is emphasised by the observation that while ferric sodium gluconate induced a number of injection-site sarcomas in mice, iron dextran glycerol glycoside (even in large and prolonged doses) showed negligible carcinogenic activity in the same species. Another feature is the apparent difference in carcinogenic potency of iron dextran glycerol glycoside in rats and mice. Although the total amount of iron administered to the mice was higher, on a body weight basis, than that given to the rats, the carcinogenic response was strikingly less. In previous investigations on macromolecular iron complexes, the response of the 2 species has usually been broadly similar.

Since different dose-schedules were used in the 2 experiments, it is not possible to compare the sarcomas induced in mice with ferric sodium gluconate, and in rats with iron dextran glycerol glycoside, in terms of their final incidence and times of induction. Histologically, however, the sarcomas were similar in the 2 groups and resembled the tumours induced by other iron-containing compounds; such lesions have frequently been described and illustrated in previous papers. One difference between rats and mice which emerged from the present study was the tendency for rats—but not mice—to develop injection-site fibromas. Fibromas are not uncommon in rats injected with macromolecular iron complexes (e.g. Roe *et al.*, 1964; Roe and Carter, 1967) but we have not seen such tumours in mice, nor are they described in other accounts dealing with the carcinogenicity of iron-compounds in mice. If this apparent species difference is a valid one, it suggests that the final neoplastic response of the subcutaneous tissues to repeated injections of iron-containing substances may be significantly different in rats and mice. Differences between rats and mice in terms of the amount of iron retained at injection sites have been reported (Golberg *et al.*, 1960; Baker *et al.*, 1960) but differences in the type of tumour produced have not been noted previously.

It is still uncertain whether iron-containing compounds are likely to induce an increase in the incidence and variety of neoplasms in tissues distant from the site of injection (Roe and Carter, 1967). But in the present study, the incidence of distant tumours in mice treated with ferric sodium gluconate, and in rats treated with iron dextran glycerol glycoside, was unusually low. Distant tumours were more numerous in mice injected with iron dextran glycerol glycoside but, as emphasised earlier, a high incidence of neoplasms was also found in the corresponding group of untreated control animals. One of the tumours encountered in a test mouse—the locally-metastasising squamous carcinoma of the forestomach—is certainly a rarity (Rowlatt, 1967) but its relationship to treatment with iron dextran glycerol glycoside is obscure.

The present findings provide more information on the carcinogenicity of iron-containing compounds in rats and mice but they do nothing towards resolving the controversy concerning the carcinogenic hazards of such compounds in man (Haddow and Horning, 1960; Baker *et al.*, 1961; Haddow *et al.*, 1964; Roe, 1966). As Haddow and his colleagues have stressed, it is still doubtful whether parenteral iron preparations have been used in clinical practice for a sufficient period of time to be certain that such materials are not carcinogenic. The therapeutic value of iron-containing compounds is beyond dispute but, at the present time, it seems reasonable to urge caution in the selection of patients and duration of treatment and to avoid the indiscriminate use of such substances.

### SUMMARY

Five out of 20 mice which received 17 once-weekly subcutaneous injections of ferric sodium gluconate (total 1 ml.) developed spindle cell or pleomorphic sarcomas at the injection site.

Ten out of 24 rats which received 25 once-weekly injections of 0·5 ml. of another proprietary preparation—iron dextran glycerol glycoside—also developed local sarcomas; in addition, 2 developed local fibromas. Of 104 mice given 5 injections of 0·1 ml., 8 injections of 0·2 ml. or 29 injections of 0·05 ml. of the same preparation, only 1 developed a neoplasm at the site of injection.

Differences between mice and rats in their response to injected iron compounds are discussed and the apparent rarity of local fibromas in mice is emphasised.

This investigation was supported by grants to the Chester Beatty Research Institute, Institute of Cancer Research: Royal Cancer Hospital from the Medical Research Council, the British Empire Cancer Campaign for Research and the Public Health Service Research Grant (CA-03188-10) from the National Cancer Institute, U.S. Public Health Service.

### REFERENCES

BAKER, S. P. DE C., GOLBERG, L., MARTIN, L. E. AND SMITH, J. P.—(1961) *J. Path. Bact.*, **82**, 453.
FIELDING, J.—(1962) *Br. med. J.*, i, 1800.
GOLBERG, L., MARTIN, L. E. AND SMITH, J. P.—(1960) *Toxic. appl. Pharmac.*, **2**, 683.
HADDOW, A. AND HORNING, E. S.—(1960) *J. natn. Cancer Inst.*, **24**, 109.

HADDOW, A., ROE, F. J. C. AND MITCHLEY, B. C. V.—(1964) *Br. med. J.*, i, 1593.
LUNDIN, P. M.—(1961) *Br. J. Cancer.*, **15**, 838.
RICHMOND, H. G.—(1959) *Br. med. J.*, i, 947.
ROE, F. J. C.—(1967) *U.I.C.C. Monograph Series*, **7**, 165.
ROE, F. J. C., HADDOW, A., DUKES, C. E. AND MITCHLEY, B. C. V.—(1964) *Br. J. Cancer*, **18**, 801.
ROE, F. J. C. AND CARTER, R. L.—(1967) *Int. J. Cancer*, **2**, 370.
ROWLATT, U. F.—(1967) In ' Pathology of Laboratory Rats and Mice '. Edited by E. Cotchin and F. J. C. Roe, Oxford (Blackwell Scientific Publications).